# Local tumor destruction and liver resection increase overall survival in intermediate/advanced hepatocellular carcinoma patients: evidence from a population-based study

**DOI:** 10.3389/fendo.2023.1191822

**Published:** 2023-07-28

**Authors:** Yang Zhang, Yi Zhang, Taiyu He, Guangliang Liu, Minjie Duan, Jian Huang, Christy Huang, Scott Lowe, Dazhi Ke, Xiaozhu Liu, Junyi Cao

**Affiliations:** ^1^ College of Medical Informatics, Chongqing Medical University, Chongqing, China; ^2^ Department of General Practice, The Second Affiliated Hospital, Chongqing Medical University, Chongqing, China; ^3^ Department of Infectious Diseases, Key Laboratory of Molecular Biology for Infectious Diseases (Ministry of Education), Institute for Viral Hepatitis, the Second Affiliated Hospital, Chongqing Medical University, Chongqing, China; ^4^ Department of Cardiology, The Second Affiliated Hospital of Chongqing Medical University, Chongqing, China; ^5^ Graduate School, Guangxi University of Chinese Medicine, Nanning, China; ^6^ College of Osteopathic Medicine, California Health Sciences University, Clovis, CA, United States; ^7^ College of Osteopathic Medicine, Kansas City University, Kansas, MO, United States; ^8^ Department of Medical Quality Control, The First People’s Hospital of Zigong City, Zigong, China

**Keywords:** liver resection, local tumor destruction, hepatocellular carcinoma, survival, nomogram

## Abstract

**Background:**

Liver resection (LR) and local tumor destruction (LTD) are effective treatments, but not commonly recommended for patients with intermediate/advanced hepatocellular carcinoma (HCC). This study aimed to explore whether LR/LTD could improve overall survival (OS) of these patients, and to identify the patients who will most likely benefit from LR/LTD.

**Methods:**

Data of patients with intermediate/advanced HCC between 2001 and 2018 were extracted from Surveillance, Epidemiology, and End Results database. OS was compared between HCC patients who received LR/LTD and those who did not. A nomogram was constructed for predicting OS, and it was then validated.

**Results:**

A total of 535 eligible patients were included, among which 128 received LR/LTD while 407 did not. Significantly higher OS in patients who received LR/LTD was observed (P<0.001). Based on independent prognostic factors obtained from univariate and multivariate analyses, a nomogram was constructed. The C-indices of nomogram were higher than those of the TNM staging system (training cohort: 0.74 vs. 0.59; validation cohort: 0.78 vs. 0.61). Similarly, areas under receiver operating characteristic curves and calibration curves indicated good accuracy of the nomogram. Decision curve analysis curves revealed good clinical practicability of the nomogram. Furthermore, low-risk patients (nomogram score: 0-221.9) had higher OS compared with high-risk patients (nomogram score: higher than 221.9) (P<0.001).

**Conclusion:**

LR/LTD significantly improves OS in patients with intermediate/advanced HCC. The nomogram developed in the present study shows high predicating value for OS in patients with intermediate/advanced HCC, which might be useful in selecting patients who are most suitable for LR/LTD.

## Introduction

With approximately 906,000 new cases and 830,000 deaths globally, primary liver cancer is the sixth most commonly diagnosed cancer and the third leading cause of cancer death in 2020 (1). Hepatocellular carcinoma (HCC) is the dominant type of liver cancer, leading to around 75% of all liver cancer cases (2). Due to the insidious onset, many patients were diagnosed with HCC at the intermediate/advanced stage. Considerable progresses have been made in the HCC treatment over the past years, and the current guidelines of the American Association for the Study of Liver Diseases (AASLD) and the European Association for the Study of Liver (EASL) recommend the transarterial chemoembolization (TACE), transarterial radioembolization (TARE), liver transplantation and systemic treatment [tyrosine kinase inhibitors (TKIs), PD-1 inhibitors] for intermediate/advanced HCC (3–5). However, the outcomes of patients with intermediate/advanced HCC were still unsatisfactory. Thus, better treatment strategies are greatly needed for outcome improvement.

Liver resection (LR) and local tumor destruction [LTD, including heat-radio-frequency ablation (RFA), etc.] were usually recommended as first-line treatment modality only for early-stage HCC, but not for intermediate/advanced HCC (3–5). As the effective improvement of survival in patients with early HCC after LR/LTD (surgery) have been reported (6, 7), it would also be of clinical significance to explore whether LR/LTD could improve the prognosis of patients with intermediate/advanced HCC, and to identify which intermediate/advanced HCC patients are the best candidates for LR/LTD from the prognostic standpoint.

In this study, eligible patients with intermediate/advanced HCC [primary tumor (T) 3/4] were included from the Surveillance, Epidemiology, and End Results (SEER) Program of the National Cancer Institute (8). The overall survival (OS) of the patients who received LR/LTD was compared with that of the patients who did not. A nomogram was constructed and validated to predict the OS of the patients to help predict which patients would be most suitable for LR/LTD.

## Methods

### Patient inclusion

**Figure 1:**
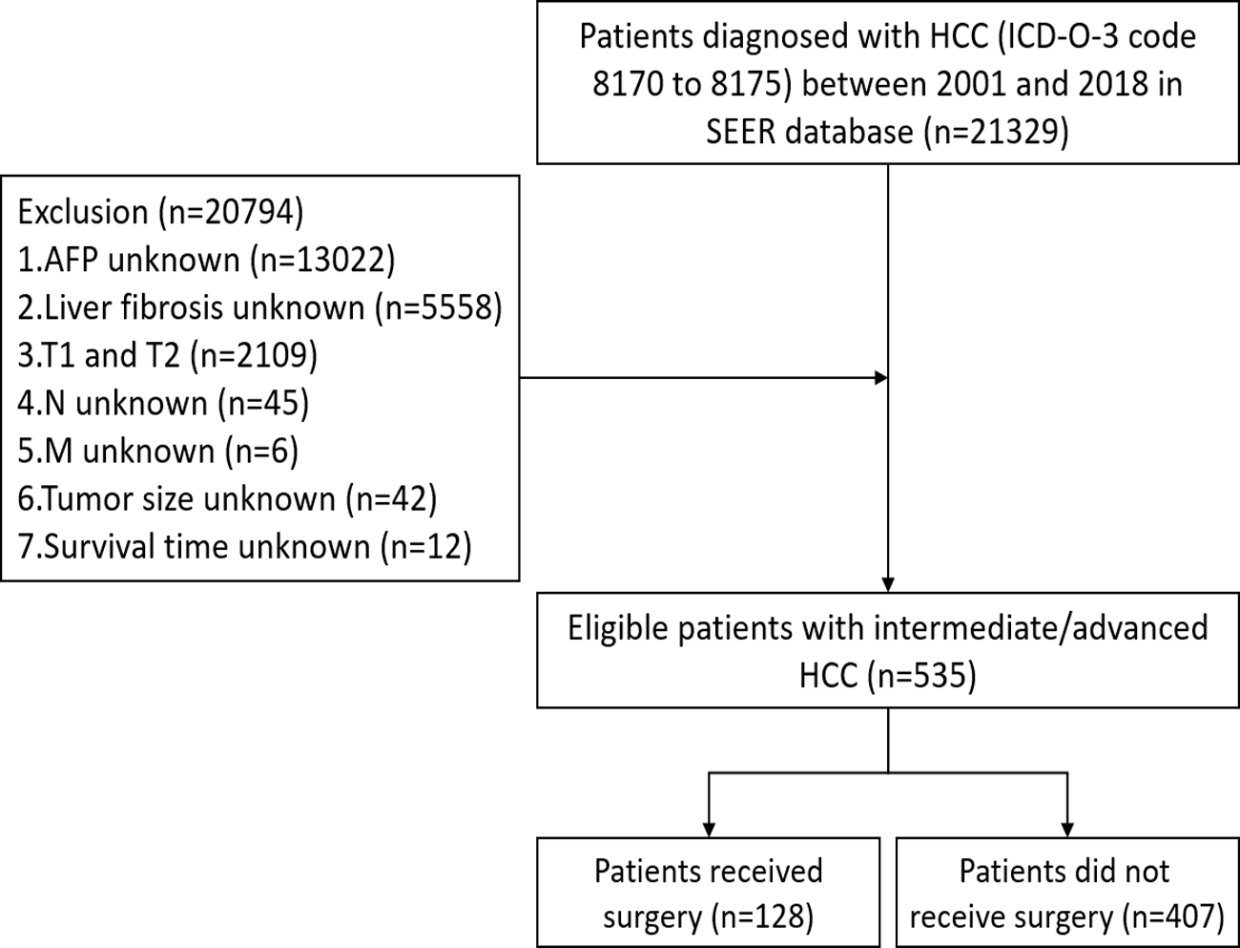
Flowchart of patient inclusion.

Data of eligible patients from 2001 to 2018 were extracted from SEER database using SEER^∗^Stat software (version 8.3.8). The inclusion criteria were as follows: 1) International Classification of Diseases for Oncology, 3rd Edition (ICD-O-3) code 8170 to 8175; and 2) T3/T4. The exclusion criteria were as follows: 1) race unknown; 2) histological grade unknown; 3) tumor size unknown; 4) regional lymph nodes (N) unknown; 5) distant metastasis (M) unknown; 6) alpha fetoprotein (AFP) unknown; 7) liver fibrosis unknown; and 8) survival time unknown. After screening, a total of 535 eligible patients with intermediate/advanced HCC were eventually included in this study (**Figure 1**). The institutional review board approval and written patient consent were not required for this study, as the data for all patients are deidentified in the publicly available SEER database.

### Statistical analysis

Propensity score matching (PSM) was performed with 1:1 nearest neighbor method to balance the characteristics of the patients who received LR/LTD (surgery) and those who did not. Chi-square test and Mann-Whitney U test were used to compare the difference of characteristics between the two groups. Kaplan–Meier curves and log-rank tests were applied for survival comparison.

Univariate and multivariate Cox proportional hazards regression analyses were used to obtain independent factors that significantly affected OS. The obtained independent factors were used for the nomogram construction. The concordance index (C-index) and receiver operating characteristic (ROC) curve were applied to reflect the discrimination and predictive accuracy of the nomogram, and their values range from 0 and 1.0. 1.0 represents perfect predicting accuracy (9). Calibration curves using a bootstrap approach with 1,000 resamples were used to compare the predicted survival with the observed survival (10). Decision curve analysis (DCA) was used to assess the clinical practicability of the nomogram (11). The cutoff value was obtained based on the log-rank test results, and then based on cutoff value, patients were divided into low-risk and high-risk groups.

R software (4.1.2, R Core Team) and relevant packages (“Matching,” “nonrandom,” “tableone,” “plyr,” “rms,” “DynNom,” “nomogramFormula,” “survival,” “foreign,” “survivalROC,” “ggDCA,” “survminer,” “glmnet,” “riskRegression”) were used for statistical analyses and plotting. The cutoff value was obtained using log-rank test. A two-sided *P*-value < 0.05 was considered statistically significant.

## Results

### Patient characteristics

The characteristics of the 535 eligible patients with intermediate/advanced HCC were summarized in **Table 1**. Of these, 128 (23.9%) patients received LR/LTD (surgery), while 407 (76.1%) patients did not. Most (85.8%) patients were at T3 stage, while 14.21% of them were at T4. 83.36% of patients were at N0, and 79.63% of them were at M0. Then, PSM was performed to balance the characteristics between the patients who received surgery and the patients who did not. Finally, 118 patients who received surgery and 118 patients who did not were enrolled for further analysis (**Table 2**).

**Table 1:** Characteristics of patients with intermediate/advanced HCC before PSM.

Variables	Overall (n = 535)	Surgery No (n = 407)	Surgery Yes (n = 128)	*P*
**Age (years), median (IQR)**	63.00 (57.00, 71.00)	63.00 (57.00, 71.00)	65.0 (56.75, 72.00)	0.59
**Race, n (%)**				0.04
Black	95 (17.76)	70 (17.20)	25 (19.53)	
White	326 (60.93)	259 (63.64)	67 (52.34)	
Other^#^	114 (21.31)	78 (19.16)	36 (28.12)	
**Sex, n (%)**				0.09
Female	112 (20.93)	78 (19.16)	34 (26.56)	
Male	423 (79.07)	329 (80.84)	94 (73.44)	
**Grade, n (%)**				<0.01
I	83 (15.51)	71 (17.44)	12 (9.38)	
II	154 (28.79)	107 (26.29)	47 (36.72)	
III	103 (19.25)	68 (16.71)	35 (27.34)	
IV	5 (0.93)	2 (0.49)	3 (2.34)	
Unknown	190 (35.51)	159 (39.07)	31 (24.22)	
**T, n (%)**				0.03
T3	459 (85.79)	357 (87.71)	102 (79.69)	
T4	76 (14.21)	50 (12.29)	26 (20.31)	
**N, n (%)**				<0.01
N0	446 (83.36)	324 (79.61)	122 (95.31)	
N1	89 (16.64)	83 (20.39)	6 (4.69)	
**M, n (%)**				<0.01
M0	426 (79.63)	303 (74.45)	123 (96.09)	
M1	109 (20.37)	104 (25.55)	5 (3.91)	
**Radiotherapy, n (%)**				0.31
No	424 (79.25)	318 (78.13)	106 (82.81)	
Yes	111 (20.75)	89 (21.87)	22 (17.19)	
**Chemotherapy, n (%)**				<0.01
No	264 (49.35)	176 (43.24)	88 (68.75)	
Yes	271 (50.65)	231 (56.76)	40 (31.25)	
**AFP, n (%)**				0.29
Positive	409 (76.45)	316 (77.64)	93 (72.66)	
Negative	126 (23.55)	91 (22.36)	35 (27.34)	
**Fibrosis, n (%)**				<0.01
Ishak 0–4	179 (33.46)	110 (27.03)	69 (53.91)	
Ishak 5–6	356 (66.54)	297 (72.97)	59 (46.09)	
**Tumor size (millimeter), median (IQR)**	80.00 (60.50, 110.00)	80.00 (60.00, 109.50)	79.00 (61.75, 110.00)	0.86

^#^Other includes Asian/Pacific Islander, American Indian/Alaskan Native. Mann-Whitney U test and Chi-square test were used for comparison. IQR, interquartile range; PSM, propensity score matching.

**Table 2:** Characteristics of patients with intermediate/advanced HCC after PSM.

Variables	Overall (n = 236)	Surgery No (n = 118)	Surgery Yes (n = 118)	*P*
**Age (years), median (IQR)**	64.00 (56.75, 73.25)	64.00 (57.00, 75.00)	64.50 (56.00, 71.75)	0.26
**Race, n (%)**				0.67
Black	41 (17.37)	18 (15.25)	23 (19.49)	
White	129 (54.66)	67 (56.78)	62 (52.54)	
Other^#^	66 (27.97)	33 (27.97)	33 (27.97)	
**Sex, n (%)**				0.45
Female	60 (25.42)	27 (22.88)	33 (27.97)	
Male	176 (74.58)	91 (77.12)	85 (72.03)	
**Grade, n (%)**				0.95
I	20 (8.47)	9 (7.63)	11 (9.32)	
II	89 (37.71)	47 (39.83)	42 (35.59)	
III	66 (27.97)	32 (27.12)	34 (28.81)	
IV	5 (2.12)	2 (1.69)	3 (2.54)	
Unknown	56 (23.73)	28 (23.73)	28 (23.73)	
**T, n (%)**				1.00
T3	190 (80.51)	95 (80.51)	95 (80.51)	
T4	46 (19.49)	23 (19.49)	23 (19.49)	
**N, n (%)**				0.49
N0	227 (96.19)	115 (97.46)	112 (94.92)	
N1	9 (3.81)	3 (2.54)	6 (5.08)	
**M, n (%)**				1.00
M0	227 (96.19)	114 (96.61)	113 (95.76)	
M1	9 (3.81)	4 (3.39)	5 (4.24)	
**Radiotherapy, n (%)**				0.34
No	187 (79.24)	90 (76.27)	97 (82.20)	
Yes	49 (20.76)	28 (23.73)	21 (17.80)	
**Chemotherapy, n (%)**				0.89
No	156 (66.10)	77 (65.25)	79 (66.95)	
Yes	80 (33.90)	41 (34.75)	39 (33.05)	
**AFP, n (%)**				0.45
Positive	178 (75.42)	92 (77.97)	86 (72.88)	
Negative	58 (24.58)	26 (22.03)	32 (27.12)	
**Fibrosis, n (%)**				0.08
Ishak 0–4	104 (44.07)	45 (38.14)	59 (50.00)	
Ishak 5–6	132 (55.93)	73 (61.86)	59 (50.00)	
**Tumor size (millimeter), median (IQR)**	78.50 (61.00, 109.00)	78.00 (61.00, 107.75)	79.50 (61.75, 110.00)	0.84

^#^Other includes Asian/Pacific Islander, American Indian/Alaskan Native. Mann-Whitney U test and Chi-square test were used for comparison. IQR, interquartile range; PSM, propensity score matching; AFP,Alpha-fetoprotein; TNM, tumor node metastasis classification.

### Surgery improved the survival of patients with intermediate/advanced HCC

**Figure 2:**
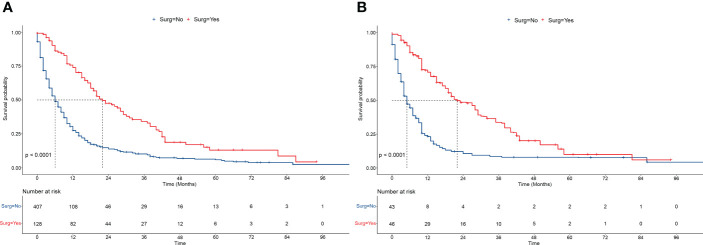
Overall survivals of patients receiving/not receiving surgery. **(A, B)** The overall survivals of patients receiving/not receiving surgery (LR/LTD) before PSM **(A)**, and after PSM **(B)**. Log-rank tests were used for survival comparison. LR, liver resection; LTD, local tumor destruction; PSM, propensity score matching.

The OS were compared between the patients with intermediate/advanced HCC who received surgery and those who did not. Before PSM, the OS was significantly higher in patients with intermediate/advanced HCC who received surgery than in patients who did not (median months: 21.0 vs. 5.0, *P*<0.001) (**Figure 2A**). The 1-, 3-, and 5-year OS rates were all higher in patients who received surgery than in patients who did not (1-year: 73.9% vs. 27.5%, 3-year: 34.3% vs. 10.2%, 5-year: 13.0% vs. 5.9%, respectively). Similarly, after PSM, the OS (median months: 21.0 vs. 4.0, *P*<0.001) (**Figure 2B**), and the 1-, 3-, and 5-year OS rates were all higher in patients who received surgery than in patients who did not.

### Univariate and multivariate analyses for independent prognostic factors

Univariate and multivariate analyses were performed to obtain the factors that significantly affected the OS. The variable was included in the multivariate analyses when its *P*-value was < 0.01. The results showed that grade, M, surgery (LR/LTD), radiation, chemotherapy, AFP, and tumor size significantly affected the OS of patients with intermediate/advanced HCC (**Table 3**).

**Table 3:** Univariate and multivariate analyses for OS-related factors.

Variables	Univariate analysis		Multivariate analysis	
	HR (95%CI)	P	HR (95%CI)	P
**Age (year)**	1.00 (0.99-1.01)	0.567	/	
Race				
Black	reference			
White	1.23 (0.92-1.65)	0.170	/	
Other^#^	1.14 (0.80-1.62)	0.481	/	
Sex				
Female	reference			
Male	0.92 (0.70-1.23)	0.589	/	
Grade				
I	reference		reference	
II	1.12 (0.78-1.63)	0.536	1.08 (0.74-1.59)	0.679
III	1.52 (1.02-2.26)	0.039	1.54 (1.01-2.35)	**0.047**
IV	1.11 (0.34-3.58)	0.867	1.66 (0.52-5.76)	0.411
unknown	1.48 (1.03-2.13)	0.033	1.28 (0.82-1.66)	0.215
T				
T3	reference			
T4	1.30 (0.93-1.82)	0.120	/	
N				
N0	reference		reference	
N1	1.60 (1.19-2.14)	0.002	1.20 (0.86-1.66)	0.282
M				
M0	reference		reference	
M1	2.08 (1.59-2.71)	<0.001	1.85 (1.38-2.49)	**<0.001**
Surgery				
No	reference		reference	
Yes	0.46 (0.35-0.61)	<0.001	0.31 (0.22-0.44)	**<0.001**
Radiotherapy				
No	reference		reference	
Yes	0.59 (0.44-0.78)	<0.001	0.46 (0.34-0.62)	**<0.001**
Chemotherapy				
No	reference		reference	
Yes	0.81 (0.64-1.01)	0.059	0.49 (0.38-0.62)	**<0.001**
AFP				
Positive	reference		reference	
negative	0.71 (0.55-0.94)	0.015	0.71 (0.52-0.95)	**0.021**
Fibrosis				
0-4	reference		reference	
5-6	1.39 (1.08-1.77)	0.009	1.23 (0.95-1.61)	0.123
**Tumor size (millimeter)**	1.00 (1.00-1.00)	0.052	1.00 (1.00-1.01)	**0.010**

^#^Other includes Asian/Pacific Islander, American Indian/Alaskan Native. CI, confidential interval; HR, hazard ratio; OS, overall survival.

### Construction and validation of the nomogram

**Figure 3:**
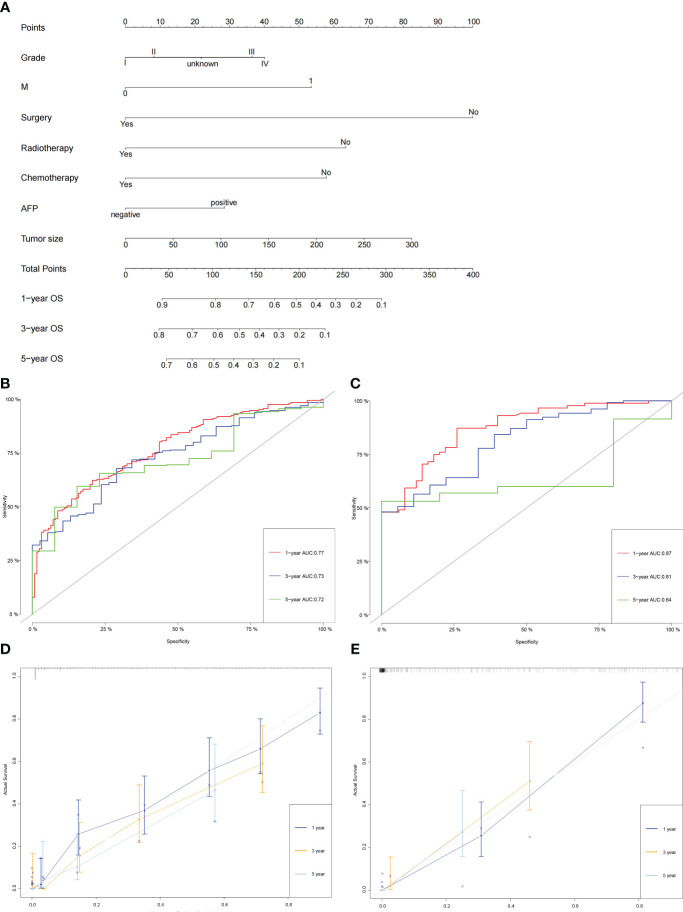
Construction and validation of the nomogram. **(A)** The nomogram for predicting the 1-, 3-, and 5-year OS of patients with intermediate/advanced HCC. **(B, C)** The 1-, 3-, and 5-year AUCs of the nomogram in the training cohort **(B)** and validation cohort **(C)**. **(D, E)** Calibration curves of the nomogram for 1-, 3-, and 5-year OS in the training cohort **(D)** and validation cohort **(E)**.

The independent prognostic factors above were included in the nomogram for predicting the 1-, 3-, and 5-year OS of patients with intermediate/advanced HCC (**Figure 3A**). To construct and validate the nomogram, 70% (n = 380) and 30% (n = 155) of all included patients were randomly assigned to the training and validation cohorts, respectively (**Supplementary Tables 1, 2**). The C-indices of the nomogram were higher than those of the American Joint Committee on Cancer (AJCC) TNM staging system (training cohort: 0.74 vs. 0.59; validation cohort: 0.78 vs. 0.61). The 1-, 3-, and 5-year areas under the ROC curves (AUCs) of the nomogram were 0.77(95%CI:0.72-0.82), 0.73(95%CI:0.66-0.80), and 0.72(95%CI:0.61-0.83), respectively, in the training cohort (**Figure 3B**). The 1-, 3-, and 5-year AUCs in the validation cohort were 0.87(95%CI:0.81-0.92), 0.81(95%CI:0.71-0.91), and 0.64(95%CI:0.50-0.78), respectively (**Figure 3C**). Similarly, the calibration curves showed moderate-to-high consistency between the nomogram predictions and the actual survivals both in training and validation cohorts (**Figures 3D, E**). Furthermore, the DCA curves demonstrated that the nomogram tended to have better positive net benefits than the TNM staging system, suggesting good clinical practicability (**Supplementary Figure 1**).

### Overall survival in low-risk and high-risk patient groups

**Figure 4:**
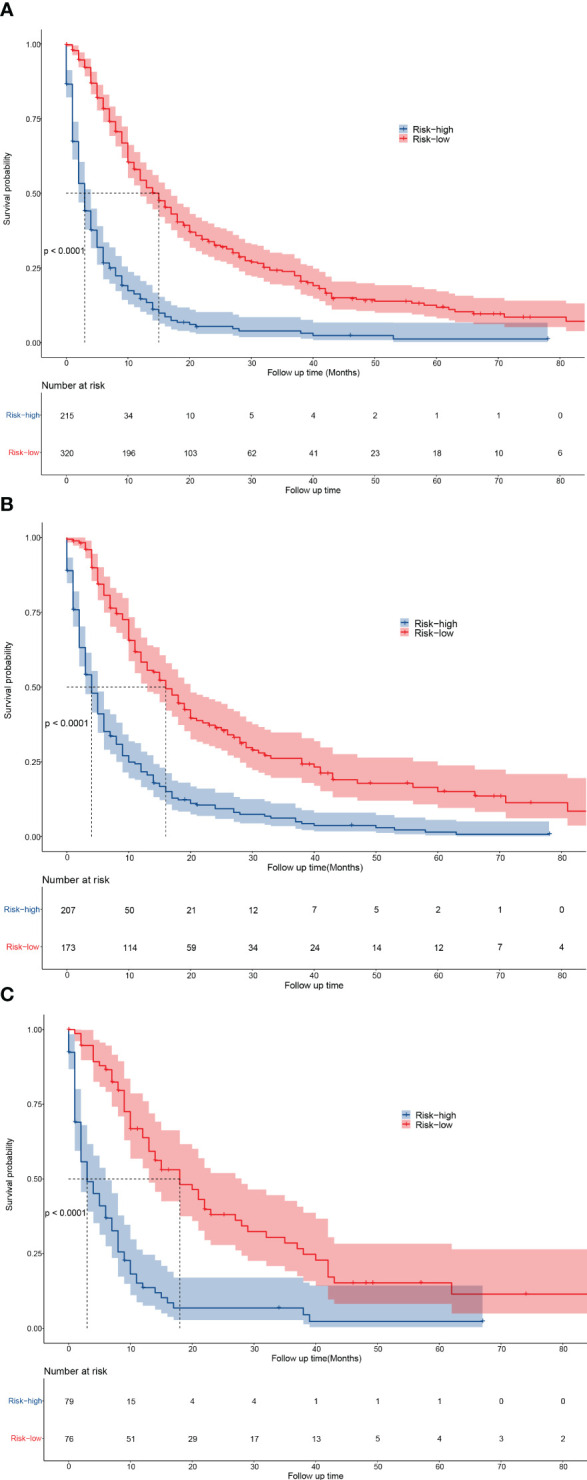
Kaplan-Meier curves for predicting the OS of low- and high-risk patients. **(A–C)** The OS of low- and high-risk patients in all cohort **(A)**, training cohort **(B)**, and validation cohort **(C)**.

According to the log-rank test results of OS, patients were divided into a low-risk group (nomogram scores ranged from 0 to 221.9) and a high-risk group (nomogram scores higher than 221.9). Kaplan–Meier curves revealed that the OS were significantly higher in the low-risk group than in the high-risk group (*P* <0.001) (**Figures 4A–C**). And the 1-, 3-, and 5-year OS rates were all higher in the low-risk group than in the high-risk group in all cohort, training cohort, and validation cohort (**Supplementary Table 3**). The results indicated good discrimination and clinical practicability of our nomogram.

### Web application for OS prediction

Based on the Nomogram prediction model constructed in this study, we have developed and deployed a web-based application for predicting the overall survival of patients with intermediate/advanced liver cancer. The application can be accessed at https://advancedlivercancer.shinyapps.io/DynNomapp/. By entering the clinicopathological characteristics of the patient, the application immediately provides the predicted survival probability for the current patient. It is designed to be simple to operate and easy to understand.

## Discussion

This study mainly explored whether surgery (LR/LTD) could significantly improve the OS of patients with intermediate/advanced HCC. Furthermore, a nomogram was constructed and validated to predict the OS of the patients, which could help to identify the patients who would most likely benefit from LR/LTD.

Previous studies have shown that both LR and LTD improved the OS and disease-free survival of patients with HCC, especially those with early HCC (6, 7). LTD is usually an alternative to LR. To date, the guidelines of AASLD and EASL still recommend LR and LTD as first-line treatment for patients with early-stage HCC, but not for patients with intermediate/advanced HCC (3–5). However, the guidelines of the Asian Pacific Association for the Study of the Liver (APASL) (12) and China (13) both point out that LR could be performed in some eligible patients with intermediate/advanced HCC. Moreover, some studies have shown that LR improved the survival in selected patients with advanced HCC (14, 15). Similarly, in this study, LR/LTD was proved to significantly improve the OS of patients with intermediate/advanced HCC, indicating good clinical benefits. Furthermore, surgery (LR/LTD) was identified to be the most important prognostic factor in our nomogram, which also suggested that receiving surgery could significantly improve the OS of patients with intermediate/advanced HCC. Though considerable progresses have been made in the HCC treatment in recent years, patients with intermediate/advanced HCC still have poor prognosis. Hence, LR/LTD might be used in certain patients to improve their OS.

A nomogram is a reliable predictive tool to calculate and predict individual survival by integrating cancer-related prognostic factors (10, 11). The results of C-indices, AUCs, calibration curves in training and validation cohorts all indicated good predictive accuracy of the nomogram developed in this study, which is better than that of the TNM staging system. Similarly, the DCA curves suggested that this nomogram tended to have better clinical practicability than the TNM staging system. Therefore, the nomogram constructed in the present study could provide great clinical value in prognostic evaluation, and assist clinicians and patients in the therapeutic strategy selection.

To further explore the values of the nomogram, the low-risk patients and high-risk patients were categorized based on their nomogram scores. It revealed that the OS was significantly higher in low-risk patients than in high-risk patients. Our results also suggested that patients with certain characteristics (low grade, M0, radiotherapy received, chemotherapy received, negative AFP, and small tumor size), namely the patients with low risk, may have good survival when receiving surgery. Therefore, surgery (LR/LTD) might be an appropriate alternative treatment in the eligible patients with intermediate/advanced HCC.

This study had several advantages. First, this is the first study to explore the effect of LR/LTD in the OS of patients with intermediate/advanced HCC in a relatively large cohort. Second, a nomogram with good accuracy and clinical practicality was constructed to predict the OS of patients with intermediate/advanced HCC. And patients with low risk (based on the nomogram) were demonstrated to be eligible for LR/LTD, which may be helpful for clinical practice. However, limitations also existed. First, data from the SEER database did not include some clinical variables, such as liver function parameters. Second, although LR/LTD were proved to improve the OS of patients with intermediate/advanced HCC, the specific treatment strategies for different patients still needed further investigation.

In summary, this study revealed that surgery (LR/LTD) could significantly improve the OS of patients with intermediate/advanced HCC, especially the low-risk patients. Our nomogram with good accuracy and clinical practicality may be used to predict the OS of patients with intermediate/advanced HCC, and to help identify the patients who would most likely benefit from LR/LTD.

## Data availability statement

The datasets presented in this study can be found in online repositories. The names of the repository/repositories and accession number(s) can be found below: https://seer.Cancer.gov.

## Author contributions

YaZ, YiZ, TH, DK, XL, JC contributed to the idea and design. GL, MD and YaZ collected and analyzed the data. XL, JH, CH, SL, and YaZ drew the figures and tables. YiZ: Writing - original draft. DK: Writing - review & editing. JC: Writing - review & editing; investigating. All authors contributed to manuscript writing and revision. All authors have reviewed the final version of the manuscript and approved its submission.
