# Supplementary material for: Local tumor destruction and liver resection increase overall survival in intermediate/advanced hepatocellular carcinoma patients: evidence from a population-based study
Source: Front Endocrinol (Lausanne). 2023 Jul 28;14:1191822. doi: 10.3389/fendo.2023.1191822 (PMC10419194; doi:10.3389/fendo.2023.1191822)
Supplement: Supplementary file 1 [file DataSheet_1.docx]

**Liver resection and local tumor destruction improve overall survival in patients with intermediate/advanced hepatocellular carcinoma: A population-based study**

Table of contents

Supplementary results......................................................................................2

Supplementary Figure 1....................................................................................2

Supplementary Table 1.....................................................................................3

Supplementary Table 2.....................................................................................4

Supplementary Table 3.....................................................................................5


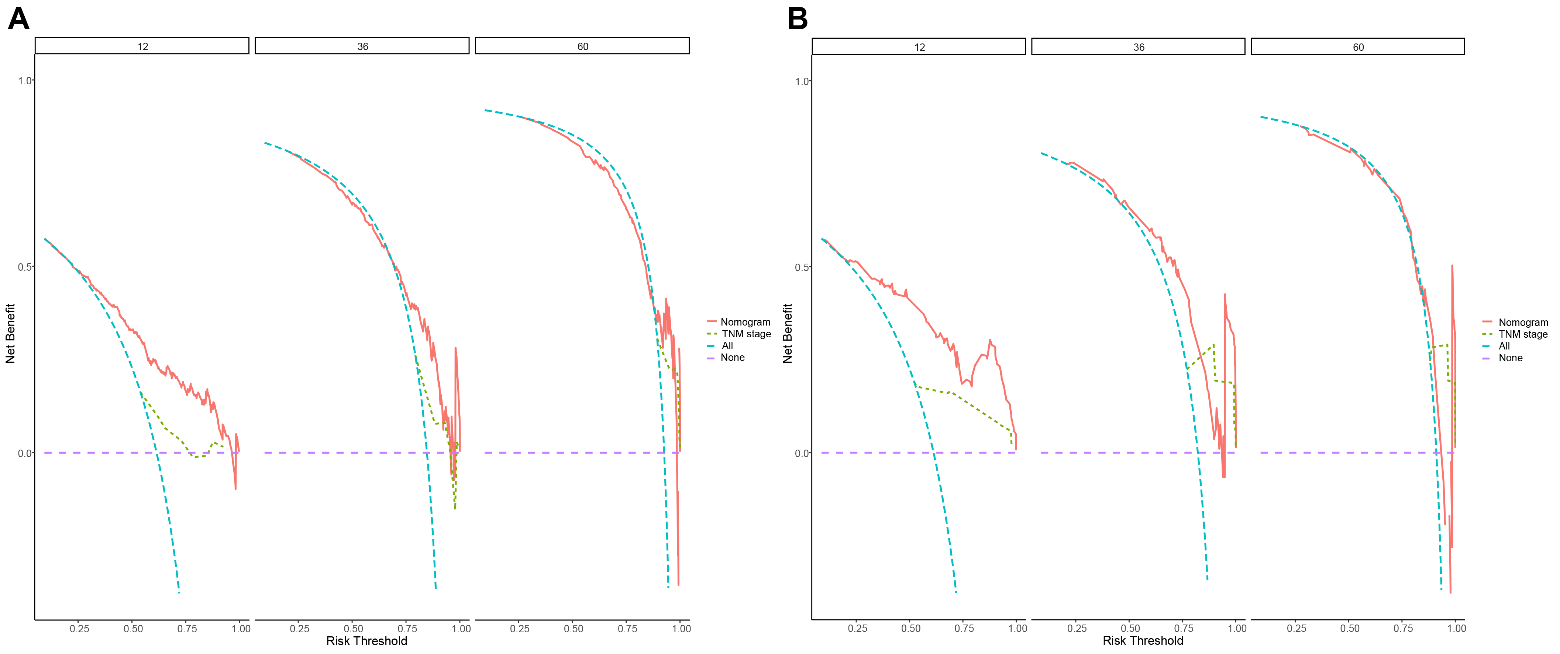


**Supplementary Figure 1. DCA curves of nomogram and TNM stage. (A, B)** DCA curves for 1-, 3-, and 5-year OS in training cohort **(A)** and validation cohort **(B)**. DCA, decision curve analysis; OS, overall survival.

| **Supplementary Table 1. Characteristics of patients in the training cohort** | | | | |
| --- | --- | --- | --- | --- |
| **Variables** | **Overall (n = 380)** | **Surgery No (n = 291)** | **Surgery Yes (n = 89)** | ***P*** |
| **Age (years), median (IQR)** | 63.00 (56.75, 70.25) | 63.00 (57.00, 70.00) | 63.00 (56.00, 75.00) | 0.603 |
| **Race, n (%)** |  |  |  | 0.138 |
| Black | 74 (19.47) | 56 (19.24) | 18 (20.22) |  |
| White | 226 (59.47) | 180 (61.86) | 46 (51.69) |  |
| Other^#^ | 80 (21.05) | 55 (18.90) | 25 (28.09) |  |
| **Sex, n (%)** |  |  |  | 0.372 |
| Female | 71 (18.68) | 51 (17.53) | 20 (22.47) |  |
| Male | 309 (81.32) | 240 (82.47) | 69 (77.53) |  |
| **Grade, n (%)** |  |  |  | 0.003 |
| I | 51 (13.42) | 43 (14.78) | 8 (8.99) |  |
| II | 113 (29.74) | 79 (27.15) | 34 (38.20) |  |
| III | 75 (19.74) | 53 (18.21) | 22 (24.72) |  |
| IV | 4 (1.05) | 1 (0.34) | 3 (3.37) |  |
| Unknown | 137 (36.05) | 115 (39.52) | 22 (24.72) |  |
| **T, n (%)** |  |  |  | 0.181 |
| T3 | 326 (85.79) | 254 (87.29) | 72 (80.90) |  |
| T4 | 54 (14.21) | 37 (12.71) | 17 (19.10) |  |
| **N, n (%)** |  |  |  | 0.002 |
| N0 | 316 (83.16) | 232 (79.73) | 84 (94.38) |  |
| N1 | 64 (16.84) | 59 (20.27) | 5 (5.62) |  |
| **M, n (%)** |  |  |  | <0.001 |
| M0 | 300 (78.95) | 216 (74.23) | 84 (94.38) |  |
| M1 | 80 (21.05) | 75 (25.77) | 5 (5.62) |  |
| **Radiotherapy, n (%)** |  |  |  | 0.569 |
| No | 297 (78.16) | 225 (77.32) | 72 (80.90) |  |
| Yes | 83 (21.84) | 66 (22.68) | 17 (19.10) |  |
| **Chemotherapy, n (%)** |  |  |  | <0.001 |
| No | 185 (48.68) | 123 (42.27) | 62 (69.66) |  |
| Yes | 195 (51.32) | 168 (57.73) | 27 (30.34) |  |
| **AFP, n (%)** |  |  |  | 0.694 |
| Positive | 294 (77.37) | 227 (78.01) | 67 (75.28) |  |
| Negative | 86 (22.63) | 64 (21.99) | 22 (24.72) |  |
| **Fibrosis, n (%)** |  |  |  | <0.001 |
| Ishak 0–4 | 119 (31.32) | 75 (25.77) | 44 (49.44) |  |
| Ishak 5–6 | 261 (68.68) | 216 (74.23) | 45 (50.56) |  |
| **Tumor size (millimeter), median (IQR)** | 80.00 (61.00, 110.00) | 80.00 (61.00, 109.50) | 80.00 (60.00, 112.00) | 0.679 |
| ^#^Other includes Asian/Pacific Islander, American Indian/Alaskan Native. Mann-Whitney U test and Chi-square test were used for comparison. IQR, interquartile range. | | | | |

| **Supplementary Table 2. Characteristics of patients in the validation cohort** | | | | |
| --- | --- | --- | --- | --- |
| **Variables** | **Overall (n = 155)** | **Surgery No (n = 116)** | **Surgery Yes (n = 39)** | ***P*** |
| **Age (years), median (IQR)** | 65.00 (58.50, 73.00) | 63.00 (59.00, 74.25) | 67.00 (58.00, 71.00) | 0.949 |
| **Race, n (%)** |  |  |  | 0.272 |
| Black | 21 (13.55) | 14 (12.07) | 7 (17.95) |  |
| White | 100 (64.52) | 79 (68.10) | 21 (53.85) |  |
| Other^#^ | 34 (21.94) | 23 (19.83) | 11 (28.21) |  |
| **Sex, n (%)** |  |  |  | 0.181 |
| Female | 41 (26.45) | 27 (23.28) | 14 (35.90) |  |
| Male | 114 (73.55) | 89 (76.72) | 25 (64.10) |  |
| **Grade, n (%)** |  |  |  | 0.013 |
| I | 32 (20.65) | 28 (24.14) | 4 (10.26) |  |
| II | 41 (26.45) | 28 (24.14) | 13 (33.33) |  |
| III | 28 (18.06) | 15 (12.93) | 13 (33.33) |  |
| IV | 1 (0.65) | 1 (0.86) | 0 (0.00) |  |
| Unknown | 53 (34.19) | 44 (37.93) | 9 (23.08) |  |
| **T, n (%)** |  |  |  | 0.115 |
| T3 | 133 (85.81) | 103 (88.79) | 30 (76.92) |  |
| T4 | 22 (14.19) | 13 (11.21) | 9 (23.08) |  |
| **N, n (%)** |  |  |  | 0.015 |
| N0 | 130 (83.87) | 92 (79.31) | 38 (97.44) |  |
| N1 | 25 (16.13) | 24 (20.69) | 1 (2.56) |  |
| **M, n (%)** |  |  |  | 0.001 |
| M0 | 126 (81.29) | 87 (75.00) | 39 (100.00) |  |
| M1 | 29 (18.71) | 29 (25.00) | 0 (0.00) |  |
| **Radiotherapy, n (%)** |  |  |  | 0.457 |
| No | 127 (81.94) | 93 (80.17) | 34 (87.18) |  |
| Yes | 28 (18.06) | 23 (19.83) | 5 (12.82) |  |
| **Chemotherapy, n (%)** |  |  |  | 0.037 |
| No | 79 (50.97) | 53 (45.69) | 26 (66.67) |  |
| Yes | 76 (49.03) | 63 (54.31) | 13 (33.33) |  |
| **AFP, n (%)** |  |  |  | 0.302 |
| Positive | 115 (74.19) | 89 (76.72) | 26 (66.67) |  |
| Negative | 40 (25.81) | 27 (23.28) | 13 (33.33) |  |
| **Fibrosis, n (%)** |  |  |  | <0.001 |
| Ishak 0–4 | 60 (38.71) | 35 (30.17) | 25 (64.10) |  |
| Ishak 5–6 | 95 (61.29) | 81 (69.83) | 14 (35.90) |  |
| **Tumor size (millimeter), median (IQR)** | 80.00 (60.00, 109.50) | 80.00 (60.00, 109.25) | 75.00 (65.00, 105.00) | 0.713 |
| ^#^Other includes Asian/Pacific Islander, American Indian/Alaskan Native. Mann-Whitney U test and Chi-square test were used for comparison. IQR, interquartile range. | | | | |

| **Supplementary Table 3. Overall survival rates of low- and high-risk patients** | | | | |
| --- | --- | --- | --- | --- |
|  |  | 1-year OS rates (%) | 3-year OS rates (%) | 5-year OS rates (%) |
| All cohort | Low-risk | 0.543 (0.489, 0.603) | 0.238 (0.191, 0.296) | 0.118 (0.081, 0.171) |
|  | High-risk | 0.146 (0.103, 0.206) | 0.039 (0.018, 0.085) | 0.012 (0.002, 0.067) |
| Training cohort | Low-risk | 0.583 (0.511, 0.665) | 0.261 (0.196, 0.348) | 0.151 (0.095, 0.238) |
|  | High-risk | 0.216 (0.165, 0.284) | 0.062 (0.035, 0.111) | 0.015 (0.004, 0.056) |
| Validation cohort | Low-risk | 0.638 (0.536, 0.760) | 0.286 (0.191, 0.427) | 0.152 (0.082, 0.283) |
|  | High-risk | 0.136 (0.075, 0.246) | 0.068 (0.027, 0.169) | 0.023 (0.004, 0.143) |
| Data are presented as median (95% CI). CI, confidential interval; OS, overall survival. | | | | |
